# Stromal VEGF-C expression predicts nodal metastasis: implications for axillary surgery de-escalation in breast cancer

**DOI:** 10.1038/s41698-026-01770-x

**Published:** 2026-11-05

**Authors:** Pauline Jeannot, Emelie Edvardsson, Oscar Hernquist, Cornelia Brandt, Linnea Fischhaber, Anna-Maria Larsson, Beatrice Öhlund, Kristian Pietras, Pär-Ola Bendahl, Charlotte Rolny, Lisa Ryden

**Affiliations:** 1 Division of Translational Cancer Research, Department of Laboratory Medicine, Lund University, Lund, Sweden; 2 Lund University Cancer Centre, Lund University, Lund, Sweden; 3 Division of Oncology, Department of Clinical Sciences, Lund University, Lund, Sweden; 4 SciLifeLab, Department of Laboratory Medicine, Lund University, Lund, Sweden; 5 Department of Gastroenterology and Surgery, Skåne University Hospital, Malmö, Sweden; 6 CIRCE: Centre for Interdisciplinary Research on Cancer and Equity in Women, Lund University, Lund, Sweden

**Keywords:** Biomarkers, Cancer, Computational biology and bioinformatics, Oncology

## Abstract

Accurate preoperative prediction of lymph node metastasis (LNM) could facilitate more individualized axillary management in breast cancer by improving identification of patients at lower risk of nodal involvement while maintaining appropriate staging for those at higher risk. Yet, reliable molecular predictors of LNM remain lacking. VEGF-C, a key driver of lymphangiogenesis, is expressed in both cancer cell and stromal compartments, but its clinical utility is unclear. In a prospectively enrolled cohort, we assessed stromal and cancer cell VEGF-C expression by immunohistochemistry using both manual scoring and a novel automated image analysis script enabling objective, reproducible classification of stromal versus epithelial cells at scale. Stromal VEGF-C was independently associated with LNM after adjustment for established predictors (*P* = 0.004), with prediction models reaching AUCs of up to 0.784. Cancer cell VEGF-C added no predictive value. In exploratory analyses, stromal VEGF-C provided predictive information comparable to that of vascular invasion, a strong predictor of LNM that is generally unavailable preoperatively. These findings identify stromal VEGF-C as a potential preoperative biomarker for nodal risk stratification. However, given the observational and predictive nature of the present study, stromal VEGF-C should be considered complementary to, rather than a replacement for, established pathological predictors. Its potential contribution to future axillary surgery de-escalation strategies warrants prospective validation.

## Introduction

Sentinel lymph node biopsy (SLNB) offers a less invasive method of axillary staging in breast cancer compared with axillary lymph node dissection (ALND), resulting in fewer complications^1^. However, SLNB is not entirely free of morbidity, as some patients still experience lymphedema, pain, or reduced arm mobility. As the proportion of patients with nodal metastases detected by SLNB has declined, recent trials such as SOUND^2^ and INSEMA^3^ have demonstrated that omission of SLNB in selected low-risk patients does not compromise oncologic outcomes, suggesting axillary surgery may be safely omitted in carefully chosen cases^2,3^. Nonetheless, reliable non-invasive methods are needed to identify patients unlikely to harbor axillary lymph node metastases  (LNM). To address this, we developed NILS (Non-Invasive Lymph Node Staging), a decision support tool that uses an artificial neural network trained on clinical and pathological data to estimate the probability of node-negative disease^4^. The model incorporates ten key clinicopathological variables, including patient factors (age and mode of detection) and tumor characteristics (tumor size, location, multifocality, histopathological type, estrogen receptor [ER] and progesterone receptor [PR] status, cancer cell proliferation [Ki-67], and vascular invasion [VI]). Upon external validation, NILS has achieved an AUC of 0.67 (95% CI: 0.62–0.72), allowing approximately 26% of patients to safely avoid axillary surgery while maintaining 90% sensitivity^5^.

Because some of the variables in the NILS model, particularly vascular invasion (VI), are inconsistently reported and/or challenging to assess preoperatively, additional biomarkers are being investigated to improve the model’s predictive accuracy. Recent evidence indicates that VEGF-C–expressing tumor-associated macrophages (TAMs) promote lymphatic rather than hematogenous spread, thereby increasing lymph node involvement^6^. Stromal VEGF-C expression may therefore be a promising biomarker for predicting LNM and enhancing non-invasive axillary staging prediction models.

## Results

### Stromal-, but not cancer cell–derived, VEGF-C is associated with lymph node metastasis by semi-quantitative scoring

**Fig. 1:**
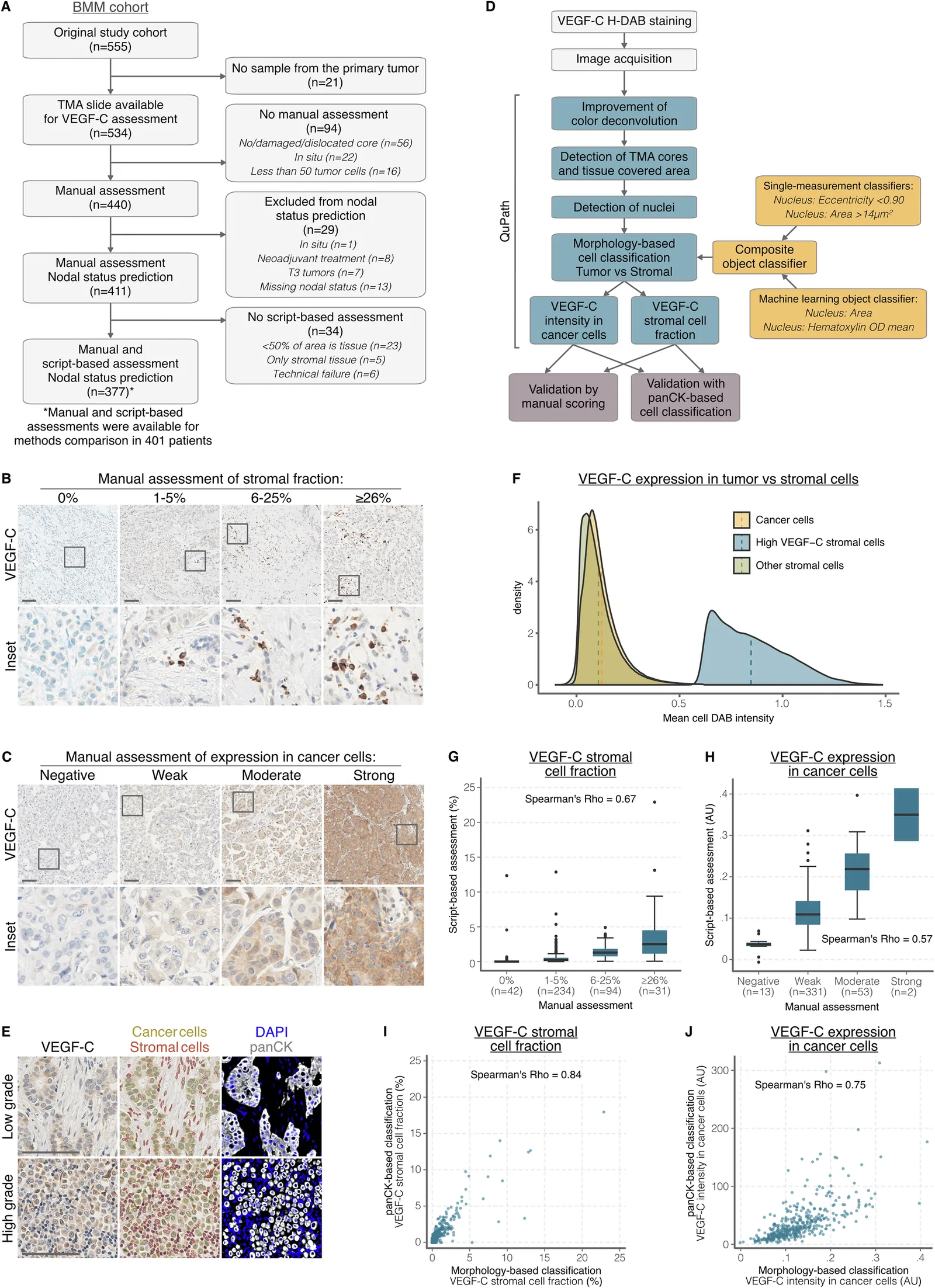
Script-based morphometric approach enables automated quantification of the VEGF-C stromal fraction and cancer-cell VEGF-C expression. **A** Flow chart illustrating the selection of patients for the Bone Marrow Metastasis cohort (BMM). **B, C** Representative immunohistochemistry images demonstrating manual scoring of the VEGF-C stromal fraction (**B**) and VEGF-C expression in cancer cells (**C**). **D** Overview of the computational pipeline used to detect VEGF-C–positive stromal cells and quantify VEGF-C expression in cancer cells. **E** Representative regions showing VEGF-C DAB staining (left), automated classification into cancer versus stromal cells (middle), and corresponding fluorescent staining for DAPI and pan-cytokeratin (panCK) (right). **F** Density plot showing the mean cell DAB intensity in the different cell populations. The dashed lines represent the mean intensity value for each group. **G, H** Boxplots comparing script-based morphometric quantification with manual assessment for the VEGF-C stromal fraction (**G**) and VEGF-C expression in cancer cells (**H**). **I, J** Dot plots comparing the VEGF-C stromal fraction (**I**) or the VEGF-C expression in cancer cells (**J**) obtained using panCK-based cell classification on fluorescent staining with the script-based morphometric approach applied to H-DAB staining. Scale bars: 100 µm.

To evaluate whether stromal and/or cancer cell derived VEGF-C expression could serve as a predictive biomarker for LNM, VEGF-C expression in both compartments was evaluated by immunohistochemistry. Figure 1A shows a flow chart illustrating the selection of patients for this project from the original 555 women enrolled in the Bone Marrow Metastasis (BMM) cohort. VEGF-C expression in stromal and cancer cells was manually quantified in primary tumor tissue microarrays (TMAs) cores from 440 patients in BMM cohort (Fig. 1B, C), of which 411 fulfilled the predefined criteria for prediction of LNM. The criteria included invasive T1-T2 tumors scheduled for primary surgery with assessable tumor tissue. Patients excluded in this step would not have been eligible for upfront SLNB. Table 1 summarizes the characteristics of this subset, overall and stratified by stromal fraction of VEGF-C. Briefly, the median age at breast cancer diagnosis was 57 years; 163 patients (40%) had node-positive disease (N+), 137 (33%) had T2 tumors, and 112 of 408 (27%) of the tumors were Nottingham histological grade (NHG) 3. VEGF-C stromal fraction was strongly associated with LNM, VI, tumor infiltrating lymphocytes (TILs), NHG, Ki-67, and age at diagnosis (Table 1). Also, VEGF-C expression in cancer cells was associated with multiple clinicopathological variables (age, VI, ER- and PR-status, Ki67, histology grade and St Gallen classification), but it was not associated with nodal status (Table 2). High-intensity VEGF-C expression was observed in stromal, but not cancer cells. VEGF-C–high stromal cells were quantified as the proportion of VEGF-C–positive stromal cells, whereas cancer cell VEGF-C showed variable intensity and was scored using four intensity classes (Fig. 1B, C).

**Table 1:** Distribution of clinicopathological characteristics in the BMM cohort and associations to manual (*n* ≤ 411) and script-based (*n* ≤ 377) assessment of fraction of VEGF-C expressing stromal cells

				VEGF-C			VEGF-C	
				Manual			Script-based, %	
	Total *n* (col %)	0% *n* (%)	1-5% *n* (%)	6-25% *n* (%)	≥26% *n* (%)	*P*-value^b^	*median (IQR)*	*P*-value^d^
No. of patients	411	49	239	90	33		≤377	
Age, years^a^	57.0 (30.1–85.4)	66.0 (42.0–81.3)	57.6 (35.1–84.4)	54.7 (33.0–75.9)	52.6 (45.1-73.8)	<0.001	−0.26^c^	<0.001
Detection						0.751		0.903
Screening	174 (42.4)	15 (8.6)	108 (62.1)	40 (23.0)	11 (6.3)		0.33 (1.10–1.20)	
Clinically detected	236 (57.6)	34 (14.4)	131 (55.5)	50 (21.2)	21 (8.9)		0.39 (0.10–1.10)	
Missing	1	0	0	0	1			
Tumor size						0.124		0.651
T1	274 (66.7)	34 (12.4)	165 (60.2)	56 (20.4)	19 (6.9)		0.39 (0.09–1.07)	
T2	137 (33.3)	15 (11.0)	74 (54.0)	34 (24.8)	14 (10.2)		0.33 (0.11–1.20)	
Tumor size, mm^a^	18 (6-50)	16 (13-22)	18 (15-25)	19 (15-27)	20 (14-25)	0.053	0.07^c^	0.159
Nodal status						0.001		<0.001
Negative	248 (60.3)	38 (15.3)	149 (60.1)	45 (18.2)	16 (6.4)		0.27 (0.05–0.85)	
Positive	163 (39.7)	11 (6.8)	90 (55.2)	45 (27.6)	17 (10.4)		0.56 (0.19–1.47)	
Multifocal						0.887		0.212
No	375 (91.2)	46 (12.3)	216 (57.6)	83 (22.1)	30 (8.0)		0.36 (0.09–1.10)	
Yes	36 (8.8)	3 (8.3)	23 (63.9)	7 (19.4)	3 (8.3)		0.45 (0.15–1.71)	
VI						0.001		<0.001
No	266 (72.1)	37 (13.9)	160 (60.2)	52 (19.6)	17 (6.4)		0.28 (0.08–0.86)	
Yes	103 (27.9)	7 (6.8)	52 (50.5)	31 (30.1)	13 (12.6)		0.57 (0.26–1.49)	
Missing	42	5	27	7	3			
TILs						<0.001		<0.001
0-9%	177 (48.0)	29 (16.4)	106 (59.9)	35 (19.8)	7 (4.0)		0.32 (0.08–0.98)	
10-49%	148 (40.1)	14 (9.5)	88 (59.5)	31 (21.0)	15 (10.1)		0.28 (0.10–0.91)	
50-74%	32 (8.7)	1 (3.1)	12 (37.5)	14 (43.8)	5 (15.6)		0.84 (0.39–1.67)	
≥75%	12 (3.2)	0 (0.0)	6 (50.0)	3 (25.0)	3 (25.0)		1.44 0.60–2.33)	
Missing	42	5	27	7	3			
NHG						< 0.001		0.194
I	85 (20.8)	14 (16.5)	52 (61.2)	16 (18.8)	3 (3.5)		0.41 (0.11–1.30)	
II	211 (51.7)	25 (11.8)	134 (63.5)	38 (18.0)	14 (6.6)		0.30 (0.09–0.91)	
III	112 (27.4)	9 (8.0)	52 (46.4)	36 (32.1)	15 (13.4)		0.48 (0.15–1.23)	
Missing	3	1	1	0	1			
ER-status						0.024		0.002
Negative	49 (12.2)	1 (2.0)	30 (61.2)	11 (22.4)	7 (14.3)		0.84 (0.26–1.89)	
Positive	353 (87.8)	48 (13.6)	203 (57.5)	77 (21.8)	25 (7.1)		0.31 (0.09–1.00)	
Missing	9	0	6	2	1			
PR-status						0.022		0.003
Negative	86 (22.7)	7 (8.1)	47 (54.6)	20 (23.3)	12 (14.0)		0.61 (0.21–1.54)	
Positive	293 (77.3)	41 (14.0)	168 (57.3)	65 (22.2)	19 (6.5)		0.30 (0.08–1.03)	
Missing	32	1	24	5	2			
HER2-status						0.220		0.447
Negative	327 (80.3)	37 (11.3)	197 (60.2)	73 (22.3)	20 (6.1)		0.34 (0.08–1.10)	
Positive	80 (19.7)	12 (15.0)	40 (50.0)	16 (20.0)	12 (15.0)		0.44 (0.15–1.13)	
Missing	4	0	2	1	1			
Ki67 ≥ 20%						< 0.001		0.029
Negative	266 (67.0)	42 (15.8)	159 (59.8)	52 (19.6)	13 (4.9)		0.32 (0.08–1.04)	
Positive	131 (33.0)	7 (5.3)	70 (53.4)	36 (27.5)	18 (13.7)		0.44 (0.15–1.21)	
Missing	14	0	10	2	2			
Histological type						0.125		0.121
NST	298 (72.7)	30 (10.1)	177 (59.4)	66 (22.2)	25 (8.4)		0.39 (0.11–1.10)	
ILC	75 (18.3)	14 (18.7)	40 (53.3)	19 (25.3)	2 (2.7)		0.27 (0.02–0.98)	
Other	37 (9.0)	5 (13.5)	22 (59.5)	5 (13.5)	5 (13.5)		0.63 (0.14–2.26)	
Missing	1	0	0	0	0			
St Gallen Classification						0.013		0.029
Luminal A/B	275 (72.6)	36 (13.1)	163 (59.3)	62 (22.6)	14 (5.1)		0.31 (0.08–0.99)	
HER2+	75 (19.8)	12 (16.0)	36 (48.0)	16 (21.3)	11 (14.7)		0.42 (0.15–1.14)	
TNBC	29 (7.6)	0 (0.0)	17 (58.6)	7 (24.1)	5 (17.2)		1.01 (0.25–2.47)	
Missing	32	1	23	5	3			

*VEGF-C* vascular endothelial growth factor C, *IQR* inter quartile range, *Multifocal* >2 cm between tumors, *VI* vascular invasion, *TILs* tumor infiltrating lymphocytes, *NHG* Nottingham histological grade, *ER* estrogen receptor >1%, *PR* progesterone receptor >1%, *HER2* human epidermal growth factor receptor 2, Ki67 a proliferation marker, *NST* no specific type, *ILC* invasive lobular carcinoma, *TNBC* triple-negative breast cancer.

^a^Median (IQR).

^b^Tests of zero slope in linear regression models with VEGF-C as the dependent variable and each of the clinicopathological variables as independent variables were used to assess evidence for association. Integer coding 1-4 was used for the four VEGF-C categories from the manual assessment. For non-ordinal variables Pearson’s Chi^2^ test was used.

^c^Spearman’s *ρ.*

^d^ Test of *ρ* = 0 for age and tumor size. Kruskal-Wallis test for categorical variables.

**Table 2:** Distribution of clinicopathological characteristics in the BMM cohort and associations to manual (*n* ≤ 411) and script-based (*n* ≤ 377) assessment of cancer cell VEGF-C intensity

		VEGF-C Manual				VEGF-C Script-based	
	Total *n* (col %)	Negative *n* (%)	Weak *n* (%)	Moderate/Strong^b^ *n* (%)	*P*-value^**c**^	median (IQR)^d^	*P*-value^e^
No. of patients	411	17	333	61		≤375	
Age, years^a^	57.0 (30.1–85.4)	58.4 (56.5–64.1)	57.8 (51.6–67.7)	53.2 (47.4–64.0)	0.010	−0.18 ^f^	<0.001
Detection					0.138		<0.001
Screening	174 (42.4)	9 (5.2)	144 (82.8)	21 (12.1)		1.68 (1.23–2.50)	
Clinically detected	236 (57.6)	8 (3.4)	189 (80.1)	39 (16.5)		2.09 (1.58–2.78)	
Missing	1	0	0	1			
Tumor size					0.284		0.038
T1	274 (66.7)	15 (5.5)	219 (79.9)	40 (14.6)		1.85 (1.33–2.60)	
T2	137 (33.3)	2 (1.5)	114 (83.2)	21 (15.3)		2.08 (1.57–2.81)	
Tumor size, mm^a^	18 (6-50)	14 (11-16)	18 (15-25)	17 (15-25)	0.269	0.12^f^	0.016
Nodal status					0.896		0.316
Negative	248 (60.3)	12 (4.8)	198 (79.8)	38 (15.3)		1.88 (1.39–2.57)	
Positive	163 (39.7)	5 (3.1)	135 (82.8)	23 (14.1)		2.05 (1.44–2.72)	
Multifocal					0.112		0.022
No	375 (91.2)	14 (3.7)	303 (80.8)	58 (15.5)		1.99 (1.44–2.71)	
Yes	36 (8.8)	3 (8.3)	30 (83.3)	3 (8.3)		1.53 (1.20–2.20)	
VI					0.041		0.008
No	266 (72.1)	11 (4.1)	221 (83.1)	34 (12.8)		1.87 (1.33–2.57)	
Yes	103 (27.9)	2 (1.9)	80 (77.7)	21 (20.4)		2.14 (1.58–2.86)	
Missing	42	4	32	6			
TILs					0.078		0.067
0-9%	177 (48.0)	10 (5.6)	142 (80.2)	25 (14.1)		1.76 (1.32–2.49)	
10-49%	148 (40.1)	3 (2.0)	124 (83.8)	21 (14.2)		2.03 (1.49–2.81)	
50-74%	32 (8.7)	0 (0.0)	26 (81.2)	6 (18.8)		2.22 (1.72–2.85)	
≥75%	12 (3.2)	0 (0.0)	9 (75.0)	3 (25.0)		1.85 (1.62–2.78)	
Missing	42	4	32	6			
NHG					0.886		0.396
I	85 (20.8)	1 (1.2)	68 (80.0)	16 (18.8)		1.85 (1.41–2.55)	
II	211 (51.7)	13 (6.2)	174 (82.5)	24 (11.4)		1.93 (1.33–2.62)	
III	112 (27.4)	3 (2.7)	89 (79.5)	20 (17.9)		1.99 (1.54–2.69)	
Missing	3	0	2	1			
ER-status					0.001		0.051
Negative	49 (12.2)	0 (0.0)	36 (70.6)	15 (29.4)		2.22 (1.63–2.83)	
Positive	353 (87.8)	17 (4.8)	288 (82.0)	46 (13.1)		1.94 (1.39–2.62)	
Missing	9	0	9	0			
PR-status					0.048		0.140
Negative	86 (22.7)	1 (1.2)	68 (79.1)	17 (19.8)		2.12 (1.55–2.74)	
Positive	293 (77.3)	16 (5.5)	237 (80.9)	40 (13.6)		1.90 (1.33–2.68)	
Missing	32	0	28	4			
HER2-status					0.284		0.312
Negative	327 (80.3)	12 (3.7)	264 (80.7)	51 (15.6)		1.95 (1.37–2.63)	
Positive	80 (19.7)	5 (6.2)	65 (81.2)	10 (12.5)		1.96 (1.57–2.68)	
Missing	4	0	4	0			
Ki67 ≥ 20%					0.004		<0.001
Negative	266 (67.0)	16 (6.0)	216 (81.2)	34 (12.8)		1.81 (1.27–2.53)	
Positive	131 (33.0)	1 (0.8)	103 (78.6)	27 (20.6)		2.21 (1.62–2.90)	
Missing	14	0	14	0			
Histological type					<0.001		0.011
NST	298 (72.7)	8 (2.7)	247 (82.9)	43 (14.4)		2.00 (1.41–2.62)	
ILC	75 (18.3)	9 (12.0)	61 (81.3)	5 (6.7)		1.70 (1.18–2.30)	
Other	37 (9.0)	0 (0.0)	25 (67.6)	12 (32.4)		2.44 (1.71–2.91)	
Missing	1	0	0	1			
St Gallen Classification					<0.001		0.059
Luminal A/B	275 (72.6)	12 (4.4)	228 (82.9)	35 (12.7)		1.94 (1.35–2.58)	
HER2+	75 (19.8)	5 (6.7)	61 (81.3)	9 (12.0)		1.96 (1.56–2.72)	
TNBC	29 (7.6)	0 (0.0)	16 (55.2)	13 (44.8)		2.49 (1.79–3.14)	
Missing	32	0	28	4			

*VEGF-C* vascular endothelial growth factor C, *IQR* inter quartile range, *Multifocal* >2 cm between tumors, *VI* vascular invasion, *TILs* tumor infiltrating lymphocytes, *NHG* Nottingham histological grade, *ER* estrogen receptor >1%, *PR* progesterone receptor >1%, *HER2* human epidermal growth factor receptor 2, Ki67 a proliferation marker, *NST* no specific type, *ILC* invasive lobular carcinoma, *TNBC* triple-negative breast cancer.

^a^ Median (IQR).

^b^ Only two patients with strong intensity, why the classes moderate and strong were merged.

^c^ Tests of zero slope in linear regression models with VEGF-C as the dependent variable and each of the clinicopathological variables as independent variables were used to assess evidence for association. Integer coding 1–4 was used for the three VEGF-C categories from the manual assessment. For non-ordinal variables Pearson’s Chi^2^ test was used.

^d^Measured in arbitrary units. Values divided by 1 SD. Hence, a difference of 1 unit between groups corresponds to a 1 SD difference.

^e^ Test of *ρ* = 0 for age and tumor size. Kruskal-Wallis test for categorical variables.

^f^ Spearman’s *ρ.*

### Novel automated QuPath-based pipeline for stromal and cancer cell classification

A composite machine-learning classifier was developed in QuPath to categorize cells as either stromal or cancer cells (Fig. 1D, E, Supplementary Fig. 1A). This classifier integrated three components: (i) a single-measurement classifier based on cell eccentricity, enabling the classification of elongated cells as stromal cells (including fibroblasts, macrophages and endothelial cells) (Supplementary Fig. 1B); (ii) a single-measurement classifier based on nuclear area, assigning cells with small nuclei to the stromal compartment (Supplementary Fig. 1C); (iii) a machine-learning classifier based on both nuclear area and nuclear hematoxylin intensity to distinguish inflammatory cells as stromal (Supplementary Fig. 1D). Following cell classification, VEGF-C intensity in cancer cells and the VEGF-C stromal cell fraction were computed for direct comparison with manual scoring. VEGF-C stromal fraction was detected using a machine-learning classifier efficiently identifying the stromal cells with strong VEGF-C expression (Fig. 1F). Concordance between manual and automated assessments was observed for stromal cell fractions (Spearman’s *ρ* = 0.67; Fig. 1G) and for cancer cell expression (Spearman’s *ρ* = 0.57; Fig. 1H). To further validate compartmental classification, dual pan-CK/VEGF-C immunofluorescence staining was performed, with pan-CK serving as a marker for cancer cells. VEGF-C intensity in cancer cells and the VEGF-C stromal cell fraction derived from this immunofluorescence approach were compared with those from the automated script-based approach, confirming accurate compartmental classification by the script-based method (Fig. 1E, I, J). However, the differences between the marker-based and morphology-based approaches highlight the limitations of morphology-based classification, which relies on cellular morphology as an indirect proxy for distinguishing stromal from cancer cells and may not accurately resolve all cellular subtypes. Furthermore, fluorescent multiplex staining of BMM cohort samples revealed that a part of VEGF-C stromal cells were co-expressing the pan-TAM marker CD68 and the endothelial marker CD31, the latter of which can also be expressed by TAMs. VEGF-C expression was not detected in CD8^+^ T cells, regardless of expression VEGF-C intensity (Supplementary Fig. 2). However, these markers do not comprehensively define the cellular identity of VEGF-C–positive stromal cells, and therefore do not allow the cellular source of stromal VEGF-C to be determined conclusively. Further work using expanded multiplex approaches will be needed to fully characterize the cellular sources of stromal VEGF-C.

**Fig. 2:**
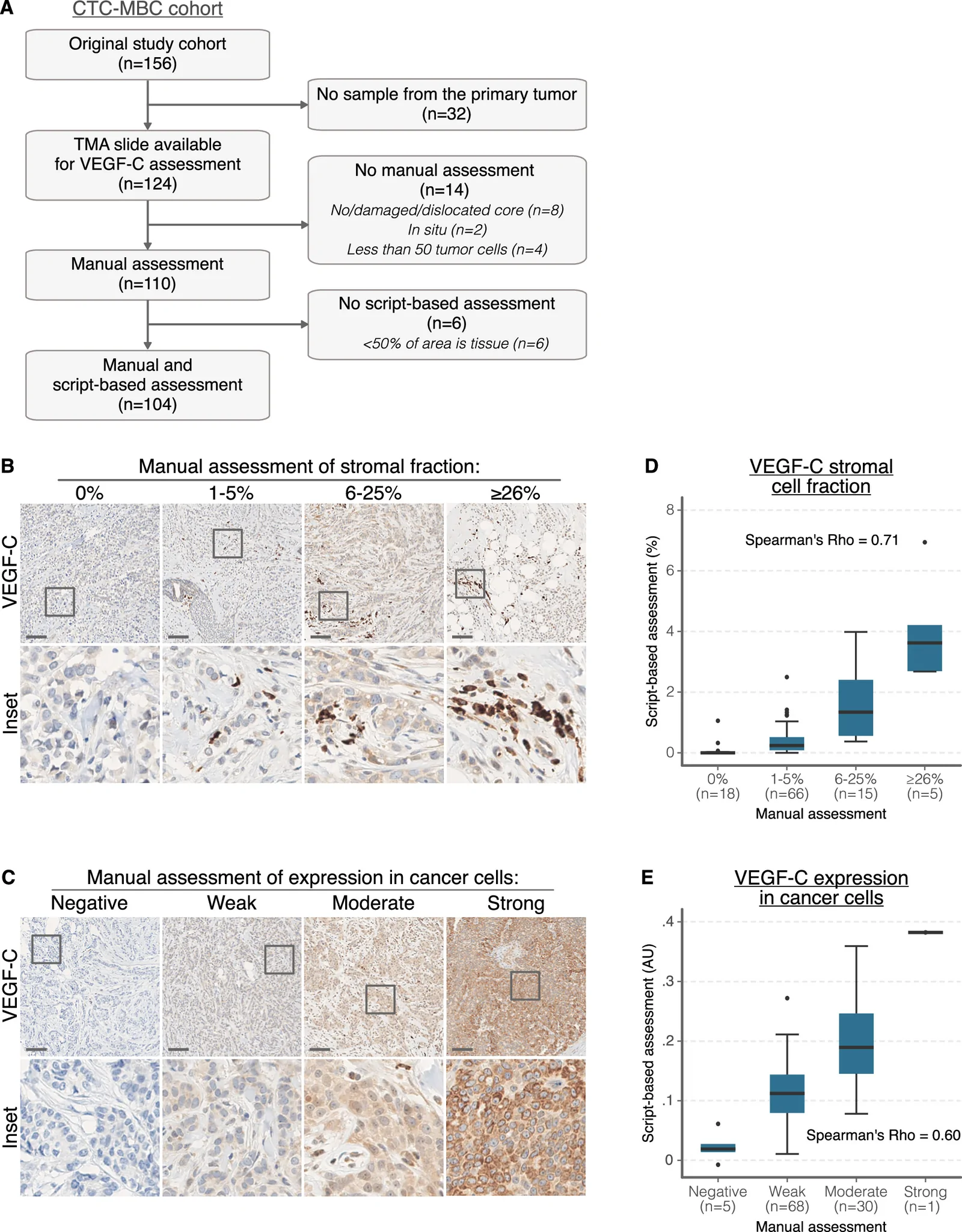
Validation of the automated morphometric approach in the CTC-MBC cohort. **A** Flow chart illustrating the selection of patients for the Circulating Tumor cells in Metastatic Breast Cancer cohort (CTC-MBC). **B, C** Representative immunohistochemistry images demonstrating manual scoring of the VEGF-C stromal fraction (**B**) and VEGF-C expression in cancer cells (**C**). **D, E** Boxplots comparing script-based morphometric quantification with manual assessment for the VEGF-C stromal fraction (**D**) or VEGF-C expression in cancer cells (**E**). Scale bars: 100 µm.

The usability of the automated script-based analysis for quantification of VEGF-C expression in stromal and cancer cells was further validated on TMAs with primary tumor samples from the circulating tumor cells in metastatic breast cancer (CTC-MBC) study. A flowchart outlining the number of patients with at least one assessable core in this cohort is presented in Fig. 2A. Consistent with the findings in the BMM cohort, automated quantification of VEGF-C expression in both stromal cells (Fig. 2B) and cancer cells (Fig. 2C) in the CTC-MBC cohort showed a strong correlation with manual scoring (Spearman’s *ρ* = 0.71 and 0.60, respectively; Fig. 2D, E). For script-based assessment in the BMM cohort, stromal VEGF-C fraction was associated with LNM (Table 1), whereas cancer cell VEGF-C intensity showed only a weak association (Table 2).

### Stromal- derived VEGF-C as a novel predictor for lymph node metastasis

Associations to LNM were further quantified as odds ratios (OR) estimated using univariable logistic regression analysis (Table 3). For manual assessment of stromal VEGF-C fraction, the category without VEGF-C expression (fraction 0%) was chosen as reference. The odds of LNM increased by a factor of 2.09 [95% CI: 1.02–4.29 *P* = 0.045, (Table 3)] for the fraction 1–5%. The corresponding multiplicative factors for the two higher classes 6–25% and ≥26% were 3.45 (95% CI: 1.57–7.60) and 3.67 (95% CI: 1.41–9.56), respectively. Also, script-based analysis of stromal VEGF-C fraction was a strong predictor of LNM [OR 1.140; 95% CI: 1.011–1.286; *P* = 0.019 (Table 3)] per percentage point. The observed relationship between the log odds of LNM and stromal VEGF-C fraction was found to be non-linear. To address this, the fractions were post-hoc transformed using log_2_. The OR per doubling of the script-based VEGF-C-fraction was 1.212 (95% CI: 1.115–1.318; *P* < 0.001; Table 3). As anticipated, also tumor size, mode of detection (clinically detected *vs* screening detected), multifocality, and VI were strong predictors of LNM (Table 3). The evidence for association between cancer cell VEGF-C intensity and LNM was, however, very weak (*P* > 0.60) for both manual and script-based assessment (Table 3).

**Table 3:** Univariable logistic regression analyses of clinicopathological variables on the outcome of positive lymph node status

	*n*	OR (95% CI)	*P-*value
Age, years	411	0.968 (0.951–0.985)	< 0.001
Detection	410		< 0.001
Screening		1.00	
Symptomatic		2.08 (1.38)	
Tumor size, mm	411	1.101 (1.071–1.132)	< 0.001
Multifocal	411		0.002
No		1.00	
Yes		2.97 (1.46–6.05)	
VI	369		< 0.001
No		1.00	
Yes		3.41 (2.13–5.48)	
NHG	408		0.086^a^
I		1.00	
II		1.16 (0.68–1.96)	0.591
III		1.80 (1.00–3.22)	0.048
ER-status	402		0.847
Positive		1.00	
Negative		1.06 (0.58–1.95)	
PR-status	379		0.854
Positive		1.00	
Negative		1.05 (0.64–1.71)	
HER2-status	407		0.269
Negative		1.00	
Positive		1.32 (0.81–2.17)	
Ki67 ≥ 20%	397		0.032
Negative		1.00	
Positive		1.59 (1.04–2.44)	
Histological type	410		0.453^a^
NST		1.00	
ILC		0.74 (0.44–1.26)	0.274
Other		0.76 (0.37–1.55)	0.451
Stromal VEGF-C fraction, manual	411		0.005^a^
0%		1.00	
1-5%		2.09 (1.02–4.29)	0.045
6-25%		3.45 (1.57–7.60)	0.002
≥26%		3.67 (1.41–9.56)	0.008
Stromal VEGF-C fraction, %, SB	377	1.140 (1.011–1.286)	0.019
Stromal VEGF-C fraction, SB log2-scale	377	1.212 (1.115– 1.318)^b^	<0.001
Cancer cells Cytoplasmic VEGF-C intensity, manual	411		0.612^a^
Negative		1.00	
Weak		1.64 (0.56–4.75)	0.365
Moderate/Strong		1.45 (0.45–4.66)	0.530
Cancer cells VEGF-C intensity, SB	375	1.04 (0.85–1.29)^c^	0.654

*n* number of patients, *OR* Odds ratio, *CI* confidence interval, *Multifocal* >2 cm between tumors, *VI* vascular invasion, *NHG* Nottingham histological grade, *ER* estrogen receptor >1%, *PR* progesterone receptor >1%, *HER2* human epidermal growth factor receptor 2, *Ki67* a proliferation marker, *NST* no specific type, *ILC* invasive lobular carcinoma, *VEGF-C* vascular endothelial growth factor C, *SB* script based.

^a^Test of overall effect: assesses whether the odds of nodal metastases are the same across all categories.

^b^Modeled on log_2_-scale; therefore, the odds ratio corresponds to a doubling of the fraction of VEGF-C-stained stromal cells.

^c^Odds ratio per one standard deviation increase in intensity (measured in arbitrary units).

As shown in Table 1, stromal VEGF-C is associated with multiple established predictors of LNM, suggesting that the crude ORs reported in Table 3 may be confounded. For example, patients lacking VEGF-C–positive stromal cells (fraction 0%, *n* = 49) had a median age of 66 years, which progressively decreased with increasing stromal VEGF-C fractions (*P*_trend_ < 0.001). To better approximate the causal association between stromal VEGF-C fraction and LNM, two multivariable logistic regression models were fitted—one per assessment method (manual scoring and script-based scoring), adjusting for identified potential confounders (Table 4). The adjusted ORs for VEGF-C were of similar magnitude to the crude ORs in Table 3, indicating that the association with LNM remained robust after adjustment for established predictors. The association was particularly strong for the script-based assessment (*P* = 0.004), supporting its potential to improve nodal prediction models.

**Table 4:** Two multivariable logistic regression analyses for positive lymph node status, including stromal VEGF-C fraction assessed manually or script-based, respectively, along with other established clinicopathological predictors of this outcome

	Manual VEGF-C			Script-based VEGF-C		
Variable	OR	95% CI	*P*-value	OR	95% CI	*P*-value
VEGF-C			0.027^a^	1.176^b^	1.055–1.311	0.004
0%	1.00					
1-5%	2.55	0.86–7.54	0.092			
6-25%	4.00	1.26–12.7	0.019			
≥26%	3.72	0.95–14.6	0.059			
Age, years	0.971	0.949–0.993	0.009	0.972	0.950–0.994	0.014
Detection						
Screening	1.00			1.00		
Clinically detected	1.35	0.79–2.30	0.272	1.29	0.76–2.20	0.349
Tumor size, mm	1.102	1.064–1.142	<0.001	1.103	1.064–1.144	<0.001
Histological type						
NST	1.00			1.00		
ILC	0.52	0.24–1.13	0.101	0.52	0.24–1.13	0.097
Other	0.62	0.23–1.69	0.348	0.54	0.20–1.47	0.228
Multifocality						
No	1.00			1.00		
Yes	3.44	1.32–8.97	0.012	3.40	1.31–8.82	0.012
VI						
No	1.00			1.00		
Yes	2.14	1.21–3.78	0.009	1.94	1.09–3.45	0.024
NHG						
I	1.00			1.00		
II	0.77	0.37–1.59	0.477	0.82	0.39–1.70	0.587
III	0.57	0.25–1.35	0.202	0.68	0.29–1.59	0.375
ER-status						
Negative	1.00			1.00		
Positive	1.26	0.54–2.94	0.594	1.42	0.61–3.33	0.417
Constant^c^	0.15	0.02–1.24		0.44	0.08–2.49	

Two complete-case analyses based on data from the same 332 patients are presented. The reduction from 377 patients, who were assessed for stromal VEGF-C fraction by both methods, is mainly due to missing data on vascular invasion (VI), a factor that is difficult to assess preoperatively.

*OR* odds ratio, *CI* confidence interval, *VEGF-C* vascular endothelial growth factor C, *NST* no special type, *ILC* invasive lobular carcinoma, *Multifocality* >2 cm between tumors, *VI* vascular invasion, *NHG* Nottingham histological grade, *ER* estrogen receptor >1%.

^a^Test of linear trend on the log-odds scale over the four manual VEGF-C categories conducted by assigning the scores 1–4 to the categories and adding this variable to the multivariable model instead of the corresponding factor variable with 3 degrees of freedom, thereby forcing the multiplicative increase in the odds of lymph node metastasis to be the same for all the three one-unit steps from 1 to 4.

^b^Modeled on log_2_-scale; therefore, the odds ratio corresponds to a doubling of the fraction of VEGF-C-stained stromal cells.

^c^The constants presented in the OR columns represent reference odds and are part of each prediction formula. They have no meaningful interpretation without centering of non-categorical variables. Hence, no *P*-values are presented.

### Integration of VEGF-C into the NILS predictive model for lymph node metastasis

We previously developed NILS, a multivariable artificial neural network (ANN)–based model for predicting LNM (4). The original model incorporated ten variables, ranked by decreasing influence: tumor size (mm), vascular invasion (VI; yes/no), multifocality (yes/no), ER status (+/−), histological type (NST/ILC/other), PR status (+/−), mode of detection (clinically detected/screening), age (years), tumor localization (four quadrants + central), and Ki67 (%).

**Fig. 3:**
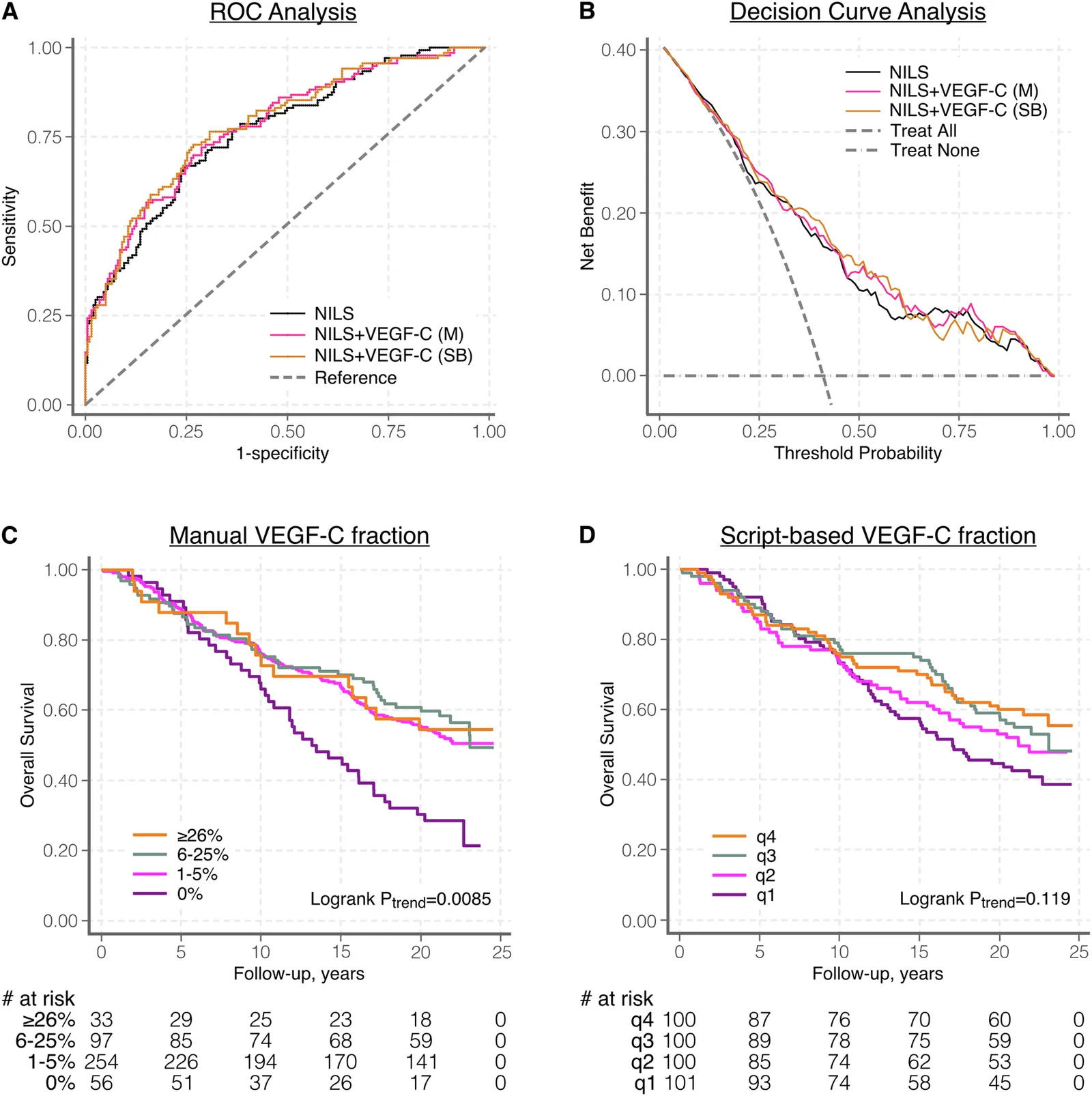
Discrimination of nodal status in multivariable prediction models with and without stromal fraction of VEGF-C, net benefit of these models, and associations with prognosis. **A** ROC-analysis comparing the discriminatory performance of three prediction models for nodal status: a reference model (NILS) and two models with stromal VEGF-C added, either manual (M) or script-based (SB) assessment. **B** Net benefit for the three models compared to the strategies of performing sentinel lymph node biopsy in all patients or in none. **C, D** Stromal VEGF-C fraction in relation to overall survival: (**C**) manually assessed (4 categories) and (**D**) script-based assessment (quartiles).

To assess the potential added predictive value of stromal VEGF-C fraction, we derived a reference model, the NILS proxy, using logistic regression in the subset of the BMM cohort with complete data for both manual and script-based VEGF-C assessment (*n* = 377). Seven of the ten original NILS variables were retained; PR and location status were excluded due to substantial missing data, and Ki67 was replaced by NHG (grades I–III), which similarly reflects tumor proliferation but is more reliably assessed. The final complete-case model was built on *n* = 332 patients, with the reduction from 377 attributable primarily to missing VI data. The NILS proxy achieved an AUC of 0.766. Incorporating semi-quantitative VEGF-C scoring modestly improved this to 0.778, and script-based quantification yielded a slight further gain, reaching an AUC of 0.784 (Fig. 3A).

To evaluate the clinical utility of VEGF-C for predicting LNM, decision curve analysis (DCA) was performed. The Net Benefit of three models was calculated across a range of threshold probabilities: the NILS-proxy model, and the NILS-proxy model augmented with stromal VEGF-C fraction, using semi-quantative and script-based assessments, respectively (Fig. 3B). All the three models have higher Net Benefit than the two extreme alternatives, SLNB for all and SLNB for none, across a wide range of clinically relevant thresholds.

A limitation of the clinical applicability of these models is that VI, one of the strongest predictors of LNM, cannot be reliably assessed on core needle biopsies^7^. We therefore examined whether stromal VEGF-C scoring could serve as a substitute for VI in nodal predictive models. The observed association between the two factors supports this hypothesis. The proportion of cases with VI increased across categories of stromal VEGF-C expression: 16% in the lowest category (0%), 25% in 1–5%, 37% in 6–25%, and 43% in ≥26%, *P* for trend = 0.001. A strong association was also observed between script-based VEGF-C assessment and VI (Mann-Whitney *P* < 0.001). Replacing VI with script-based VEGF-C fraction in the LNM prediction model yielded a larger AUC gain (ΔAUC = 0.022), bringing the AUC to 0.772. Model comparison via Akaike’s information criterion (AIC) further supported the added value of VEGF-C: its inclusion produced a greater improvement than VI alone (ΔAIC = 10.4 vs. 6.4), with the 4.0-unit difference surpassing the widely accepted threshold of 2 units for a meaningful distinction^8^. Script-based stromal VEGF-C fraction was the second most influential predictor of LNM after tumor size and enabled inclusion of patients with missing VI, increasing the sample size from 332 to 366. In this expanded cohort, the NILS proxy model without VI but with script-based stromal VEGF-C achieved an AUC of 0.779. Results for manual VEGF-C fraction were similar.

### Stromal-derived VEGF-C expression and patient´s prognosis

Since stromal VEGF-C fraction was shown to predict LNM, which is an established predictor of poor survival, we also investigated its association with overall survival. Kaplan-Meier estimates for each of the four categories of manually assessed stromal VEGF-C fraction and for quartiles of the script-based fraction are shown in Fig. 3C, D. A log-rank test for trend showed strong evidence for association to overall survival for the manual assessment (*P* = 0.0085 versus *P* = 0.119). More specifically, the survival seems to be poor in the group lacking stromal VEGF-C expression (manual fraction 0%). A post hoc univariable Cox-regression contrasting this category with the other three categories combined, was used to quantify this crude multiplicative effect on survival. The hazard ratio (HR) was 1.91 (95% CI: 1.36–2.69), however, this estimate turned out to be confounded by age. Patients lacking stromal VEGF-C were, on average, 7.0 years older than those with a fraction ≥1%, resulting in an age-adjusted HR of 1.20 (95% CI: 0.84–1.71). The association between cancer cell VEGF-C intensity and overall survival was even weaker (data not shown). Associations to the endpoints breast cancer-specific survival, recurrence-free survival and distant recurrence-free interval were in the same direction and of essentially the same magnitude (data not shown).

## Discussion

In this prospective–retrospective study of primary breast cancer patients, we demonstrate for the first time that stromal VEGF-C expression is an independent predictor of LNM, underscoring its potential as a novel biomarker for nodal staging. In contrast, VEGF-C expression in cancer cells showed no predictive value for nodal status. Importantly, script-based VEGF-C assessments yielded results comparable to manual scoring, supporting the feasibility of digital pathology for future clinical implementation. These findings are particularly relevant in the context of current efforts to de-escalate axillary surgery, and they highlight the critical importance of identifying the cellular source of a biomarker when evaluating its prognostic and clinical utility.

Over the past two decades, axillary management in breast cancer has shifted substantially, with ALND largely abandoned in favor of SLNB^9^, which currently is also under scrutiny. Updated ASCO guidelines^10^ recommend omission of SLNB in selected patients, postmenopausal women with clinical node-negativity, low-risk tumors undergoing breast-conserving surgery including radiotherapy and endocrine therapy—based on the SOUND^2^ and INSEMA trials^3^ which showed non-inferiority of omission. However, these trials excluded younger patients and those undergoing mastectomy, limiting generalizability of the findings. Consistent with ASCO guidelines, axillary omission should be individualized using risk models integrating tumor biology, patient preferences, and clinical stage. Current nodal prediction models rely mainly on clinical stage, as tumor phenotype, including ER status, adds limited value. Although VI strongly predicts nodal involvement, its limited assessment on core biopsies restricts preoperative use. Stromal VEGF-C may represent a candidate surrogate for VI, warranting further evaluation for its potential to improve preoperative nodal risk stratification and inform future strategies for axillary surgery de-escalation. Although the AUC gain from adding VEGF-C expression to the multivariable NILS model was modest, AUC is a stringent metric, particularly when the baseline model already shows good discrimination^8^. The gain of adding VEGF-C to the model was more evident when improvement was measured by AIC or a likelihood ratio test.

The finding that stromal, rather than cancer cell, VEGF-C expression predicts regional lymph node metastasis is puzzling but may be explained when  quantitative and biochemical evidence are considered together. Quantitative analysis of VEGF-C immunoreactivity across our cohorts revealed that high-expressing stromal cells exhibit approximately 6-fold greater median staining intensity than cancer cells or low-expressing stromal cells (Fig. 1F). Although high VEGF-C-expressing stromal cells are numerically fewer than cancer cells, their markedly elevated per-cell output likely generates a local concentration gradient sufficient to engage the peritumoral lymphatic endothelium in a manner that the more diffuse signal from cancer cells cannot, consistent with the well-established dose-response relationship between VEGF-C levels and lymphatic endothelial sprouting and migration^11^. The stromal cells responsible for this high-intensity signal most likely comprise TAMs as the dominant source, with additional contributions from CAFs and endothelial cells. This is consistent with prior reports linking TAM-derived VEGF-C to lymphatic microvessel density and vascular invasion in lymph node-positive breast cancer^12^ and with data demonstrating that peritumoral VEGF-C-positive TAMs correlate with lymphatic vessel invasion and lymph node metastasis^13^. In our own published work, we showed that VEGF-C-expressing TAMs redirect cancer cell dissemination toward the lymphatic route by promoting peritumoral lymphangiogenesis via VEGFR-3 signaling^6^. A further mechanistic consideration explains why cancer cell-derived VEGF-C, even when detectable by IHC, is likely to be functionally inert. VEGF-C is secreted as an inactive precursor requiring stepwise enzymatic removal of both propeptides, mediated by proteases including ADAMTS3, plasmin, cathepsin D, and thrombin, before acquiring receptor-binding competence^11,14,15^. Cancer cells appear inefficient at executing this proteolytic activation and likely depend on stromal cells to supply the requisite enzymes^16^. Compounding this, pro-VEGF-C accumulates as an inactive ECM-anchored reservoir via its C-terminal propeptide, with efficient processing requiring the stromal co-expression of ADAMTS3 and its obligate cofactor CCBE1^15^. As our antibody does not discriminate between precursor and mature isoforms, IHC positivity in cancer cells likely substantially overestimates their functional lymphangiogenic contribution. By contrast, TAMs both produce VEGF-C and co-express its activating proteases, generating the fully processed mature form capable of high-affinity VEGFR-3 binding and additional VEGFR-2 engagement^15,17^. This dual capacity for high-level production and autonomous proteolytic activation positions TAMs as uniquely equipped effectors within the tumor microenvironment, delivering bioactive VEGF-C to the peritumoral lymphatic endothelium and establishing the paracrine gradient required for lymphangiogenesis and subsequent lymphatic dissemination, an effect that the comparatively lower and predominantly unprocessed VEGF-C from cancer cells alone cannot replicate.

We show that absence of stromal VEGF-C was associated with worse survival in univariable analyses; however, this association was substantially attenuated after adjustment for age. Patients with stromal VEGF-C–negative tumors were, on average, seven years older than those with detectable stromal expression. These findings suggest that loss of stromal VEGF-C reflects age-associated remodeling of the tumor microenvironment rather than an independent prognostic determinant. Aging is known to alter stromal cell populations, including fibroblasts, endothelial cells, and TAMs, which undergo age-dependent phenotypic and functional changes^18^. Given the capacity of TAMs to produce and respond to VEGF-C, reduced stromal VEGF-C expression in older patients likely represents a feature of microenvironmental aging rather than a tumor-intrinsic driver of clinical outcome.

Key limitations include the retrospective assessment of VEGF-C in archival tumor material and the presence of non-assessable cores, which reduced the number of evaluable cases, particularly in complete-case analyses. Although the cohort was prospectively enrolled, the use of archival material for biomarker assessment may limit the direct applicability of these findings to routine preoperative tissue assessment. Future studies should evaluate larger, independent cohorts and use multiplex immunofluorescence or related spatial profiling approaches to further characterize the cellular origin and phenotype of VEGF-C–positive stromal cells. Importantly, assessment of VEGF-C in diagnostic core needle biopsy specimens will be needed to determine its feasibility and predictive value in the preoperative setting and to establish whether it can complement existing clinicopathological predictors in future axillary risk stratification.

In conclusion, stromal, but not cancer cell–derived, VEGF-C is an independent, dose-dependent predictor of lymph node metastasis in primary breast cancer. Using a novel automated script based tool on a single immunohistochemical marker, stromal VEGF-C was quantified objectively and reproducibly in a large prospectively enrolled cohort with long-term follow-up, showing strong concordance with manual scoring in an independent validation cohort. This approach reduces observer bias, supports integration into modern pathology workflows, and highlights stromal VEGF-C as an additional biomarker for preoperative nodal risk stratification. However, its clinical utility and potential contribution to axillary surgery de-escalation require prospective validation before it can be used to guide surgical decision-making.

## Methods

### Study cohorts

A total of 555 patients were included in the prospective BMM cohort, established to assess the prognostic significance of cytokeratin-positive cells in bone marrow^19^. Formalin-fixed, paraffin-embedded primary tumors were retrieved to construct TMAs^19^. Biomarker analyses included ER, PR, Ki-67, and HER2 status^20^ and vascular invasion was retrospectively scored on whole-slide sections^21^. An independent validation cohort of 156 patients from a prospective study^22^ of the CTC-MBC cohort was included for comparison of assessment protocols only^23^. Survival data were retrieved from Statistics Sweden ((https://www.scb.se/en/) in November 2024, and recurrence data were obtained from patient records. Only patients eligible for upfront SLNB (T1–T2, no neoadjuvant therapy) were included in LNM prediction analyses, while all patients with available VEGF-C scores were included in method concordance and prognostic analyses. TIL scoring was performed by a clinical pathologist on whole slide sections according to the Immuno-Oncology International TILs Working Group guidelines, in which stromal TILs are defined as the proportion of the stromal area showing lymphocytic infiltration with no direct contact with invasive tumor cells^24^.

### Ethics

All patients provided written informed consent at time of inclusion, and the studies were approved by the Ethics Committee at Lund University (LU699-09, LU75-02, and LU2010/35). Retrieval of outcome data from Statistics Sweden and individual patient files was approved in 2023 by the Swedish Ethics Review Authority (2023-01457-02 and 2023-03844-01).

### Immunohistochemistry

Paraffin-embedded TMA blocks were sectioned into 4.0 μm slices using a Microm HM355S microtome (Thermo Fisher Scientific, Kalamazoo, MI, USA). Automated immunohistochemical (IHC) staining was performed using the Ventana Discovery Ultra platform (Roche Diagnostics Corp., Indianapolis, IN, USA), including deparaffinization, antigen retrieval, and blocking of non-specific interactions. A monoclonal mouse anti-VEGF-C antibody (clone E6, sc-374628; Santa Cruz Biotechnology, Dallas, TX, USA) was used as the primary antibody, using a dilution of 1:100, incubated for 32 min at 37 °C. The secondary antibody was Discovery OmniMap HRP-conjugated anti-mouse (Roche Diagnostics, Solna, Sweden), incubated for 16 min at 37 °C. Signal detection was carried out using the ChromoMap DAB kit (Roche Diagnostics, Solna, Sweden). The protocol was optimized using tonsillar and breast tumor tissue, with lymphatic endothelial cells, mesothelial cells, reticular cells, and follicular dendritic cells serving as positive controls for VEGF-C staining. Slides containing TMA cores were scanned using a NanoZoomer S60 Digital Slide Scanner (Hamamatsu Photonics, Hamamatsu, Japan) and digitally analyzed with PathXL Xplore (Philips, Amsterdam, Netherlands) at ×20 magnification. On average, two cores were assessed per patient, and the maximum score of matching cores was used in all analyses.

### Multiplex immunofluorescence staining

Multiplex immunofluorescence (mIF) staining was performed on a Ventana Discovery Ultra automated slide stainer (Roche Diagnostics Corp., Indianapolis, IN, USA) using Opal tyramide signal amplification (TSA) chemistry (Akoya Biosciences, Menlo Park, CA, USA). Six protein targets were stained sequentially in the following order: VEGF-C, CD31 (pan-endothelial marker, with reported expression on TAMs), CD68 (pan-macrophage marker), CD8 (cytotoxic T-cells), and pan-cytokeratin (CKPAN), epithelial compartment). Nuclei were counterstained with DAPI. Full antibody details, including clone, catalog number, dilution, and assigned Opal fluorophore, are provided in RR.

Each staining cycle comprised on-board deparaffinisation, epitope retrieval, primary antibody incubation, secondary antibody/HRP multimer incubation, Opal TSA dye deposition, and heat-based stripping. Epitope retrieval was performed using Ventana Cell Conditioning Solution 1 (CC1, pH 8.0; Roche Diagnostics) at 95 °C for 64 min, unless otherwise stated. All primary antibodies in this panel were raised in mouse; detection was therefore achieved using the OmniMap anti-mouse HRP multimer (Roche Diagnostics) incubated for 16 min at 37 °C. Opal TSA fluorophore reagents were diluted 1:150 in 1× Plus Amplification Diluent (Akoya Biosciences) and applied for 16 min at 37 °C. Between cycles, antibody–HRP complexes were stripped using Ventana Cell Conditioning Solution 2 (CC2, pH 6.0; Roche Diagnostics) at 100 °C for 8 min; this step dissociates the primary–secondary–HRP complex while preserving the covalently deposited Opal fluorophore.

Detection of CKPAN (cycle 6) required a modified dual-pretreatment strategy to optimize epitope accessibility: CC1 retrieval (32 min, 95 °C) was followed by on-board enzymatic retrieval with Protease 3 (P3; Roche Diagnostics) for 4 min at 37 °C. To accommodate the Opal 780 spectral range without cross-reactivity, CKPAN signal was amplified through an intermediate TSA-DIG step (Opal detection kit 9; Akoya Biosciences), followed by application of an anti-DIG HRP conjugate (1:25, 60 min, room temperature) and subsequent Opal 780 TSA deposition (1:150, 16 min, 37 °C).

Following completion of all staining cycles, nuclei were counterstained with DAPI (3 drops/mL^−^ PBS, 8 min, room temperature). Slides were rinsed, air-dried briefly, and mounted with ProLong Gold aqueous mountant (Thermo Fisher Scientific) under No. 1.5 borosilicate cover slips. Mounted slides were stored flat in the dark at 4 °C until imaging.

#### Multispectral image acquisition

Stained slides were scanned on an Akoya PhenoImager HT multispectral imaging system (Akoya Biosciences) using a 40× objective. Whole-slide multispectral image cubes were acquired across seven fluorescence channels corresponding to DAPI, Opal 480, Opal 520, Opal 570, Opal 620, Opal 690, and Opal 780. Per-channel exposure times were determined empirically using single-stain control slides prepared alongside each staining run.

#### Spectral unmixing and quantitative image analysis

Spectral unmixing was performed in Akoya InForm software (v2.4) using a library built from single-stain controls for each Opal fluorophore and an autofluorescence reference derived from unstained FFPE tissue of the same histological type. Unmixed component images were exported for downstream analysis in *QuPath* (version 0.5.1)^25^. Cell segmentation used DAPI to define nuclear boundaries and CKPAN staining was used to classify cells as cancer or stromal cells. Regions of interest were manually curated to exclude necrotic foci, tissue folds, and section-edge artefacts prior to analysis.

### Manual scoring of VEGF-C expression

VEGF-C expression was independently assessed by two blinded evaluators using a predefined scoring protocol. A third assessor was involved in discordant cases. Exclusion criteria were missing or damaged cores, <50 invasive cancer cells, and cores containing only in situ lesions. Cytoplasmic VEGF-C staining in cancer cells was evaluated for both intensity (0 = negative; 1 = weak; 2 = moderate; 3 = strong) and fraction of positive cancer cells (0 = 0%; 1 = 1–5%; 2 = 6–25%; 3 = 26–50%; 4 = 51–75%; 5 ≥ 76%). All cancer cells had a homogenous distribution of staining in the tumor area, thus only intensity was considered for further analysis. Stromal cells, defined as non-epithelial, non-cancerous cells within the tumor microenvironment, were assessed according to the same criteria. However, since all stained stromal cells had a strong intensity, only the fraction of VEGF-C expressing stromal cells is reported in this study, and since only a single tumor had a VEGF-C fraction above 50%, the classes 3-5 were merged in all analyses. Blood vessels and muscle fibers were excluded from stromal scoring.

### Script-based scoring (QuPath)

Digital images were imported into *QuPath* (version 0.5.1)^25^. Color deconvolution was optimized for each cohort to improve separation of the hematoxylin and DAB signals. For the BMM cohort, stain vectors were set to hematoxylin (0.73111, 0.59519, 0.33351) and DAB (0.34211, 0.5857, 0.73479) with a background value of (236, 231, 227). For the CTC cohort, stain vectors were set to hematoxylin (0.71817, 0.58684, 0.37398) and DAB (0.2286, 0.53372, 0.81418) with a background value of (235, 230, 226). TMA cores were automatically identified using QuPath’s TMA dearrayer plugin (*qupath.imagej.detect.dearray.TMADearrayerPluginIJ*), with a target core diameter of 1.0 mm, a density threshold of 5, and a boundary scale of 105%. For the BMM cohort, multiple TMA slides with differing label orientations were processed using cohort-specific dearraying scripts. For the CTC cohort, a single dearraying configuration was applied. For both cohorts, manual adjustments were applied where automatic dearraying was suboptimal. Cores with >50% missing or damaged tissue, consisting solely of stromal tissue or out of focus were excluded from analysis. Tissue areas within each TMA core were segmented using the *Simple Tissue Detection* module (*qupath.imagej.detect.tissue.SimpleTissueDetection2*). Detection was performed at a pixel size of 5.0 μm with an intensity threshold of 225 on a brightfield background. Regions smaller than 10,000 μm² were excluded, holes smaller than 1000 μm² were filled, and image smoothing and median cleanup were applied prior to detection.

Nuclei detection was performed using the *Watershed Cell Detection* algorithm (*qupath.imagej.detect.cells.WatershedCellDetection*) based on the hematoxylin Optical Density (OD) channel. For the two cohorts, detection was performed at a requested pixel size of 0.5 μm, with a Gaussian sigma of 1.1 μm, a background radius of 12.0 μm (estimated by reconstruction). A hematoxylin OD threshold of 0.09 was applied to the BMM cohort and 0.15 to the CTC cohort. Detected nuclei were required to have an area between 8.0 and 150.0 μm². Cells were defined by expanding each detected nucleus by 1 μm, and all morphological measurements were recorded for downstream classification.

#### Cell compartment classification — BMM cohort

To classify detected cells as stromal or cancer cells, a composite object classifier (*Combined_Size_Ecc_Hemato*) was applied in three sequential steps. First, an artificial neural network multilayer perceptron (ANN_MLP) classifier was trained using two nuclear features — nucleus area and nucleus hematoxylin OD mean — with a symmetric sigmoid activation function and Resilient Backpropagation (RPROP) training (up to 1000 iterations, convergence threshold *ε* = 0.01). This classifier assigned cells to either a high-hematoxylin class (stromal) or tumor cell class. Second, a threshold-based classifier on nucleus eccentricity (threshold: 0.899) excluded elongated cells from the tumor cell class, reclassifying those with eccentricity ≥0.899 as non-tumor. Third, a threshold-based classifier on nucleus area (threshold: 14.0 μm²) excluded small nuclei from the tumor cell class, reclassifying those with area <14.0 μm² as non-tumor. These three classifiers were applied sequentially as a composite pipeline to all detected cells.

#### VEGF-C expression quantification — BMM cohort

For each TMA core, VEGF-C staining intensity in cancer cells was quantified from the DAB OD channel and the mean DAB OD per cancer cell was calculated. In parallel, a second ANN_MLP classifier (*VEGFC_high*) was trained on cell-level DAB OD mean to classify stromal cells as VEGF-C high or VEGF-C low, using a symmetric sigmoid activation function and RPROP training (up to 1000 iterations, *ε* = 0.01). The classifier was trained using annotation criteria aligned with manual scoring to ensure comparability between automated and manual assessments. The count of VEGF-C-high stromal cells was normalized to the total number of stromal cells per core to determine the fraction of VEGF-C expressing stromal cells.

#### Cell compartment classification — CTC cohort

For the CTC cohort, cell detection thresholds and classifier features were adapted to account for differences in tissue processing and staining. A composite classifier (*Size_Ecc_HematoX_filter*) was applied using the same three-step sequential architecture as the BMM cohort, but with an expanded feature set for the ANN_MLP step. Eight nuclear features were used: nucleus area, eccentricity, hematoxylin OD mean, sum, standard deviation, maximum, minimum, and range. The ANN_MLP used a symmetric sigmoid activation function with RPROP training (up to 1000 iterations, *ε* = 0.01). Threshold-based filters were subsequently applied for nucleus eccentricity (threshold: 0.923) and nucleus area (threshold: 14.0 μm²), using the same logic as the BMM cohort.

#### VEGF-C expression quantification — CTC cohort

VEGF-C expression in stromal cells of the CTC cohort was quantified using a dedicated ANN_MLP classifier (*VEGFC_high*) trained on six nuclear DAB OD features: mean, sum, standard deviation, maximum, minimum, and range. This extended feature set, compared to the single DAB OD mean used in the BMM cohort, was employed to improve classification specificity given the different staining characteristics of the CTC tissue. The classifier used a symmetric sigmoid activation function with RPROP training (up to 1000 iterations, *ε* = 0.01) and output cells as VEGF-C high or VEGF-C low. The classifier was trained using annotation criteria aligned with manual scoring to ensure comparability between automated and manual assessments. The fraction of VEGF-C expressing stromal cells was calculated as in the BMM cohort.

All QuPath scripts and trained classifiers for both the BMM and CTC cohorts, including color deconvolution settings, TMA dearraying configurations, tissue and cell detection parameters, composite cell classification pipelines, and VEGF-C scoring classifiers, have been deposited as described under the Code Availability section.

### Statistical methods

Associations between the manually assessed fraction of VEGF-C-expressing stromal cells and clinicopathological variables were summarized using median and range for continuous variables and percentages for categorical or of no association to VEGF-C was evaluated using a test of zero slope in a linear regression of the variable on the categorical integer coded VEGF-C variable, i.e., a test for linear trend across VEGF-C categories. A *χ*² test was applied for the non-ordinal St Gallen Classification. Logistic regression was used to evaluate associations with the binary outcome LNM. The joint predictive value of variables in multivariable logistic regression models was assessed using ROC-analysis, and discrimination was summarized as AUC (area under the ROC curve). Net Benefit was evaluated using decision curve analysis, and prognostic value was examined using Kaplan-Meier survival estimates, log-rank tests and Cox proportional hazards regression. All statistical tests presented are two-sided. P-values, which were not adjusted for multiple comparisons, should be interpreted as measures of evidence rather than compared to a fixed threshold for significance. Stata 19.0 (StataCorp LLC, TX, USA) was used for all the statistical calculations.

**Research Resource Identifiers**

**Table:** 

Reagents/Instruments/Software	Source	Identifier
anti-VEGF-C antibody (clone E6)	Santa Cruz Biotechnology	Cat# sc-374628; RRID:AB_11012156
anti-panCK antibody (clone: AE1/3)	Agilent Technologies	Cat #M3515; RRID:AB_2132885
anti-CD68 antibody (clone: PG-M1)	Agilent Technologies	Cat #M0876; RRID: AB_2074844
anti-CD8 antibody (clone C8/144B)	Agilent Technologies	Cat #M7103; RRID:AB_2075537
anti-CD31 antibody (clone JC70)	Roche Diagnostics	Cat #760-4378; RRID:AB_2927455
OmniMap HRP-conjugated anti-mouse	Roche Diagnostics	Cat# 760-4310; RRID:AB_2885182
Ventana DISCOVERY ultra	Roche Diagnostics	Cat# 750-601; RRID:SCR_021254
Cell Conditioning 1 (CC1)	Roche Diagnostics	Cat# 950-500
ChromoMap DAB Kit (RUO), DISCOVERY	Roche Diagnostics	Cat# 760-159
Hematoxylin II	Roche Diagnostics	Cat# 790-2208
NanoZoomer S60 Digital Slide Scanner	Hamamatsu Photonics	RRID:SCR_022537
PhenoImager HT	Akoya	RRID:SCR_023772
QuPath	https://qupath.github.io	RRID:SCR_018257
R	https://www.r-project.org	RRID:SCR_001905
RStudio	https://posit.co/	RRID:SCR_000432
STATA	StataCorp LLC	RRID:SCR_012763

## Supplementary information

Supplementary Figures

## Data Availability

The data that support the findings of this study are not openly available due to reasons of sensitivity and are available from the corresponding author upon reasonable request. Data are located in controlled access data storage at Lund University.

## Code availability

All scripts used in this study have been deposited at [https://github.com/rolnylab/Jeannot-Edvardsson-BreastCancer-Stromal-VEGFC LymphNode/tree/main], DOI link found at [https://doi.org/10.5281/zenodo.18803748]
