# Supplementary material for: Stromal VEGF-C expression predicts nodal metastasis: implications for axillary surgery de-escalation in breast cancer
Source: NPJ Precis Oncol. 2026 Nov 5;10:428. doi: 10.1038/s41698-026-01770-x (PMC13740231; doi:10.1038/s41698-026-01770-x)
Supplement: Supplementary file 1 [file 41698_2026_1770_MOESM1_ESM.pdf]

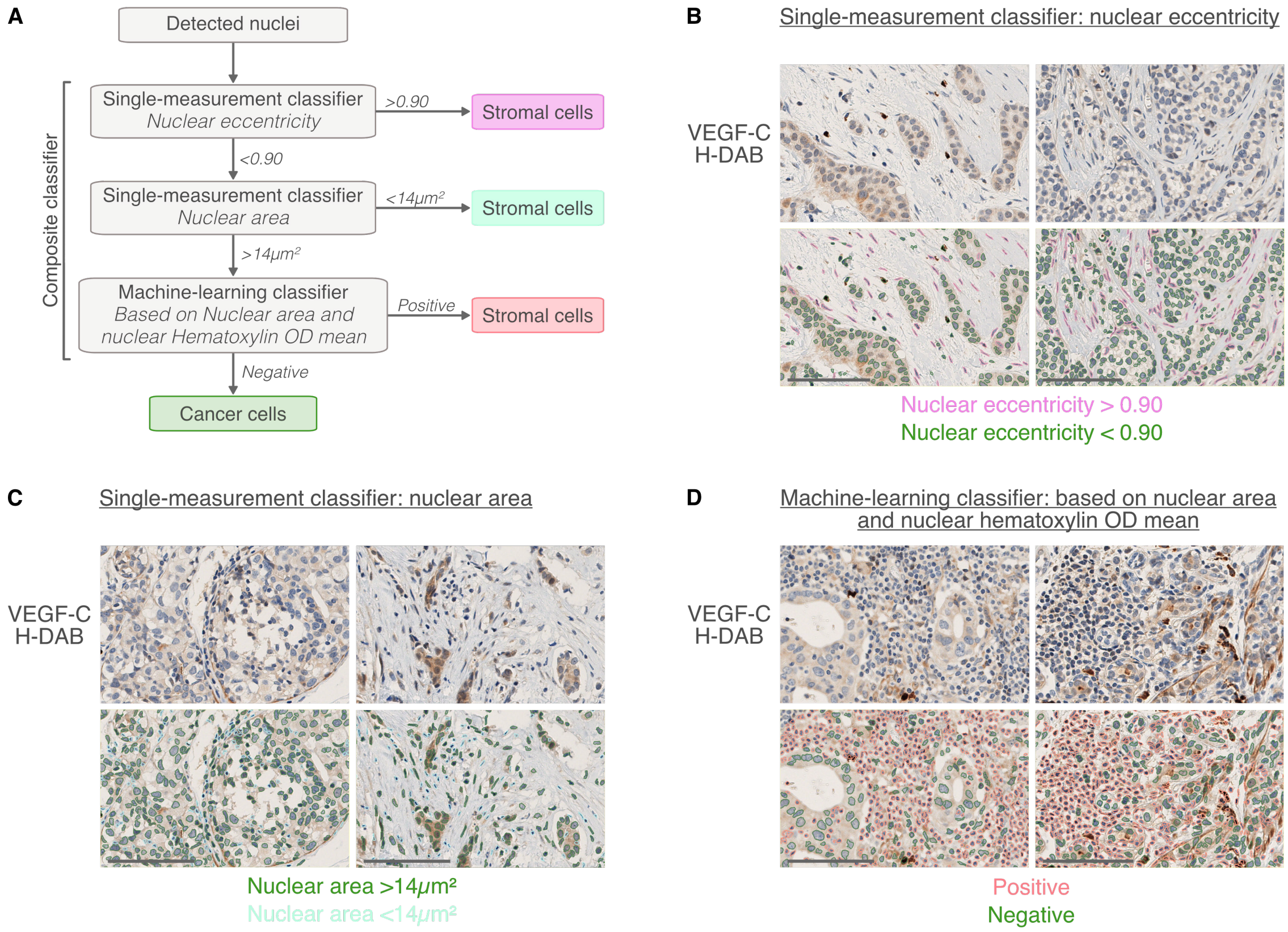

Supplementary Figure 1: Composite classifier for discrimination of stromal and cancer cells in H-DAB-stained sections.

(**A**) Flowchart illustrating the components of the composite classifier and their sequential contribution to cell identity assignment. (**B-D)** Representative images showing classifications performed by the single-measurement classifier based on nuclear eccentricity **(B)**, the single-measurement classifier based on nuclear area **(C)**, and the machine-learning classifier based on nuclear area and nuclear hematoxylin optical density (OD) mean **(D)**. Scale bars: 100μm

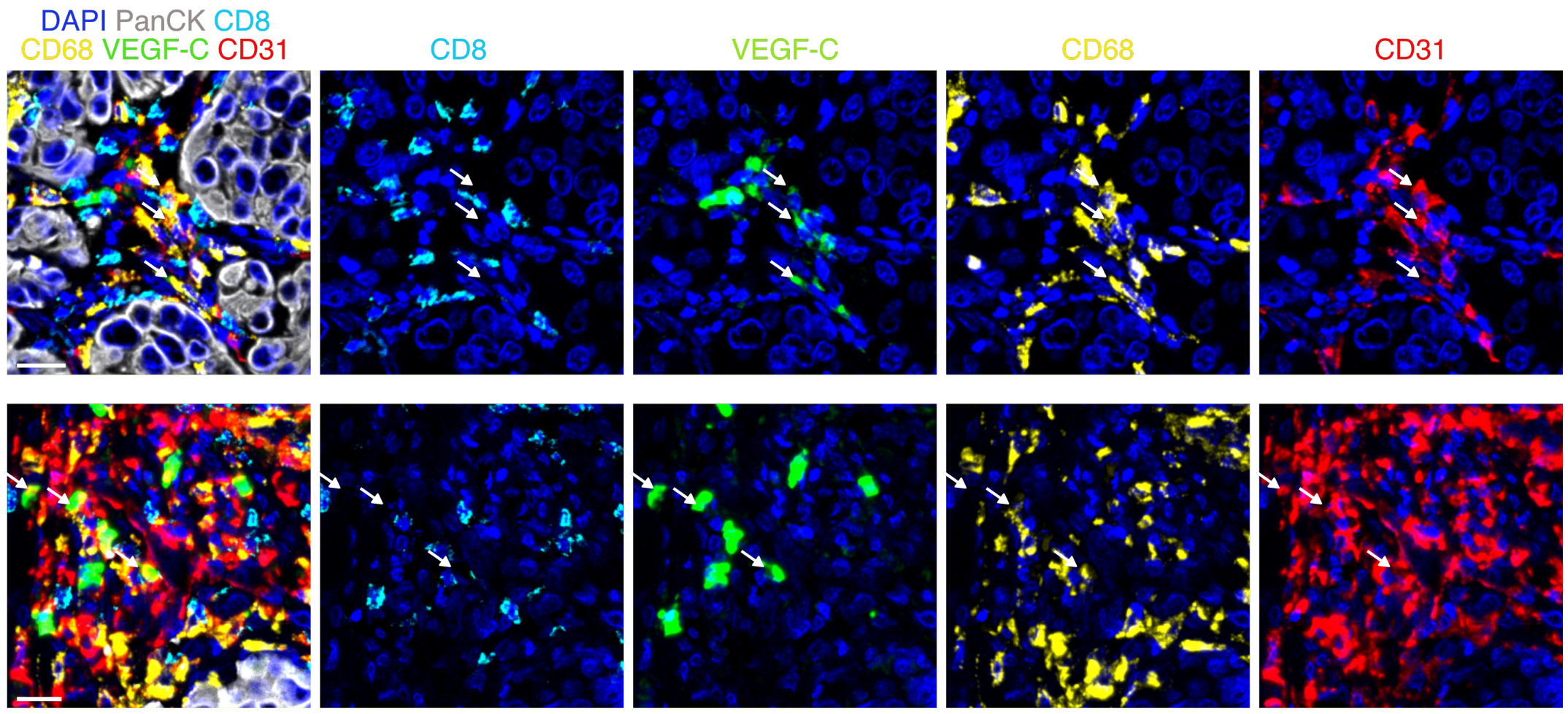

Supplementary Figure 2: Characterization of VEGF-C stromal cells by multiplex immunofluorescence.
Example images of multiplex immunofluorescent staining in BMM cohort samples, using a panel comprising pan-CK, CD8, VEGF-C, CD68 and CD31. Arrows indicate stromal cells co-expressing VEGF-C with CD68 and/or CD31. Scale bars: 20μm
